# Characterization of c-Src During *Listeria monocytogenes* Cell-to-Cell Spreading

**DOI:** 10.1093/infdis/jiaf063

**Published:** 2025-02-03

**Authors:** Petra A McLeod, Aaron S Dhanda, Julian A Guttman

**Affiliations:** Department of Biological Sciences, Centre for Cell Biology, Development, and Disease, Simon Fraser University, Burnaby, British Columbia, Canada; Department of Biological Sciences, Centre for Cell Biology, Development, and Disease, Simon Fraser University, Burnaby, British Columbia, Canada; Department of Biological Sciences, Centre for Cell Biology, Development, and Disease, Simon Fraser University, Burnaby, British Columbia, Canada

**Keywords:** endocytosis, Src-dependent phosphorylation, bacterial dissemination, host-pathogen interactions, *Listeria monocytogenes*

## Abstract

*Listeria monocytogenes* replicates within host cells and spreads from cell to cell using actin-based motility. Cell-to-cell movement of *L. monocytogenes* is achieved by creating actin-rich membrane protrusions (listeriopods), which generate corresponding invaginations in adjacent cells through caveolin-mediated endocytosis. We show that c-Src, a multifunctional tyrosine kinase, is enriched at invaginations and is crucial for efficient cell-to-cell spreading of the bacteria as cells expressing c-Src mutants that were either constitutively active or those that impeded the function of c-Src resulted in significantly more (or less) cell-to-cell spreading. This work demonstrates the importance of c-Src in influencing *L. monocytogenes*’ ability to spread intercellularly.

## Teaser

This study describes the role of c-Src at *Listeria monocytogenes*-generated invaginations and its impact on cell-to-cell spreading of the bacteria.

*Listeria monocytogenes* is a gram-positive, food-borne pathogen that causes listeriosis, killing approximately 25% of people it infects [1]. Fundamental for *L. monocytogenes* infectivity is its ability to enter cells and spread from cell to cell. Initial invasion begins through the expression of the bacterial proteins internalin A and B that bind to host E-cadherin and c-Met receptors, respectively [2, 3]. Clathrin-mediated endocytosis is then used to internalize the bacteria briefly enclosed in vacuoles that they escape through the expression of the pore-forming toxin listeriolysin O and phospholipases [4]. In the cytoplasm, *L. monocytogenes* become motile by hijacking the actin cytoskeleton of their host cells through the expression of ActA [5], which recruits and activates the Arp2/3 complex by mimicking N-WASp, forming actin-rich comet tails [6].

Crucial to *L. monocytogenes*’ infections are their ability to spread from one cell to another. This is accomplished by *L. monocytogenes* actin comet tails pushing against the host cell's plasma membrane creating actin-rich finger-like protrusions (called listeriopods). These listeriopods push into neighboring (receiving) cells, creating corresponding invaginations, which ultimately engulf the bacterium within double membrane vacuoles. Listeriopod internalization in recipient cells has been shown to act through the actions of caveolin-mediated endocytosis [7, 8].

As this endocytic mechanism requires Src-mediated tyrosine phosphorylation in other systems, we explored the role of c-Src during *L. monocytogenes* cell-to-cell spreading. C-Src is a multifunctional tyrosine kinase with a conserved architecture consisting of SH2 (binding to proline rich motifs), SH3 (binding to tyrosine residues), SH4 (catalytic kinase domain), and N-terminal myristylation (required for membrane attachment) domains. The only nonconserved domain is the unique domain, which was shown to regulate the SH3 and SH4 domains [9].

C-Src phosphorylates caveolin-1 and dynamin-2 to promote swelling or fission of the caveolar complex [10, 11]. Thus, we explored the potential roles of c-Src in the cell-to-cell transfer of *L. monocytogenes.* We found that upregulated or downregulated (or domain deletions of) c-Src caused an accompanying increase or decrease in *L. monocytogenes* cell-to-cell spreading.

## METHODS

### Cell Culture

Jeg3, HeLa (American Type Culture Collection) and HeLa caveolin-1 knockdown (cav-1 KD) cells were cultured using Minimum Essential Medium (MEM) and Dulbecco's Modified Eagle Medium (DMEM) with 10% fetal bovine serum (FBS) (Gibco). HeLa cav-1 KD cell lines were generated as previously described [7] supplemented with puromycin (1 µg/mL). Cells were maintained at 37°C and 5% CO_2_. The cells were washed with phosphate-buffered saline without Ca2+ and Mg2+(PBS−/−), (Gibco), trypsinized with 0.05% trypsin-EDTA (Gibco), and seeded into 6- or 24-well culture plates with or without glass coverslips.

### Transfections

DNA transfections involved combining 1.5 μg DNA, 3 μL JetPRIME, and JetPRIME buffer to create a 100-μL solution. Cells were transfected in serum-free MEM/DMEM at 37°C. Medium was replaced with MEM/DMEM containing 10% FBS after 4 hours and incubated for at least 24 hours at 37°C for gene expression.

### *L. monocytogenes* Infections

Green fluorescent protein (GFP) or wild-type *L. monocytogenes* (EGD BUG 2539/600) were grown on brain heart infusion (BHI) plates (37°C). A single colony was grown overnight in 2 mL of BHI broth in a shaking incubator (37°C). Overnight cultures were diluted 2:1 in BHI broth and incubated (37°C). When the optical density 600 = 1.00, 1 mL of bacteria was pelleted (25°C), then washed with PBS−/−. Repelleted bacteria were resuspended in MEM (Jeg-3) or DMEM (HeLa) (37°C), then diluted 1000 times in media and added into culture plates containing host cells.

### Immunolocalization

Samples were fixed with 3% paraformaldehyde and permeabilized using 0.2% Triton X-100 in PBS−/−. After blocking with 5% normal goat serum, samples were incubated with primary antibodies (anti-Src, 2 µg/mL; anti-hemagglutinin tagged [HA], 8 µg/mL; Invitrogen) at 4°C overnight. Cells were stained with AlexaFluor 488 or 594 secondary antibodies and AlexaFluor 405 or 594 phalloidin (Invitrogen). After washing with PBS−/−, samples were mounted onto glass slides with Prolong Diamond antifade mounting medium (with or without 4′,6-diamidino-2-phenylindole [DAPI]). Imaging was performed using a Leica DMI4000B microscope.

### Cell-to-Cell Spreading Assays

Jeg-3 cells were seeded at a density of 1 × 10^6^ into two 6-well culture dishes without coverslips and 1 population of cells was transfected. On day 3, nontransfected cells were infected with GFP-*L. monocytogenes*. After 2 hours, medium containing 10% FBS and 50 µg/mL of gentamycin was added to each well. After 3 hours, approximately 300 000 of the transfected cells and approximately 20 000 of the infected cells were added to 24-well plates containing MEM with 10% FBS and 50 µg/mL gentamycin. The combined cells were centrifuged at 10 000 rpm for 3 minutes and incubated at 37°C for 6 hours. The samples were then fixed and stained with mouse anti-HA antibodies (described above).

### Lysate Preparation and Western Blotting

Cells were lysed in radioimmunoprecipitation assay lysis buffer containing protease inhibitors (Roche), scraped off the plates, and centrifuged. Supernatant was collected, and protein concentrations were measured using a bicinchoninic acid assay kit (Pierce). Lysates with 6× sample buffer were boiled for 10 minutes and vortexed. Equal amounts of protein were loaded onto 10% sodium dodecyl sulfate-polyacrylamide gels and resolved by electrophoresis. After transferring the proteins onto nitrocellulose membrane, samples were blocked with 4% Blotto and stained overnight with primary antibodies at 4°C. Membranes were washed with Tris-buffered saline plus Tween and subjected to secondary antibodies (horseradish peroxidase-conjugated goat anti-rabbit or anti-mouse antibodies, 1 µg/mL; Invitrogen). Membranes were treated with Western Lightning Plus-ECL (PerkinElmer) and imaged on a Fujifilm LAS-4000 imager. Membranes were stripped and reprobed using mouse anti–α-tubulin antibodies (1:500; 12G10, Developmental Studies Hybridoma Bank).

### Statistical Analysis

Immunofluorescence was performed in triplicate (n = 3) with at least 30 fields of view for actin-rich structures. Src mutants were normalized to wild type. Statistical analysis was performed using 1-way ANOVA (*P* < .01) in GraphPad Prism. Line scan analysis was done in ImageJ Fiji with the Plot Profile plugin, measuring the pixel intensities of a specific channel across a line.

## RESULTS

**Figure 1.**
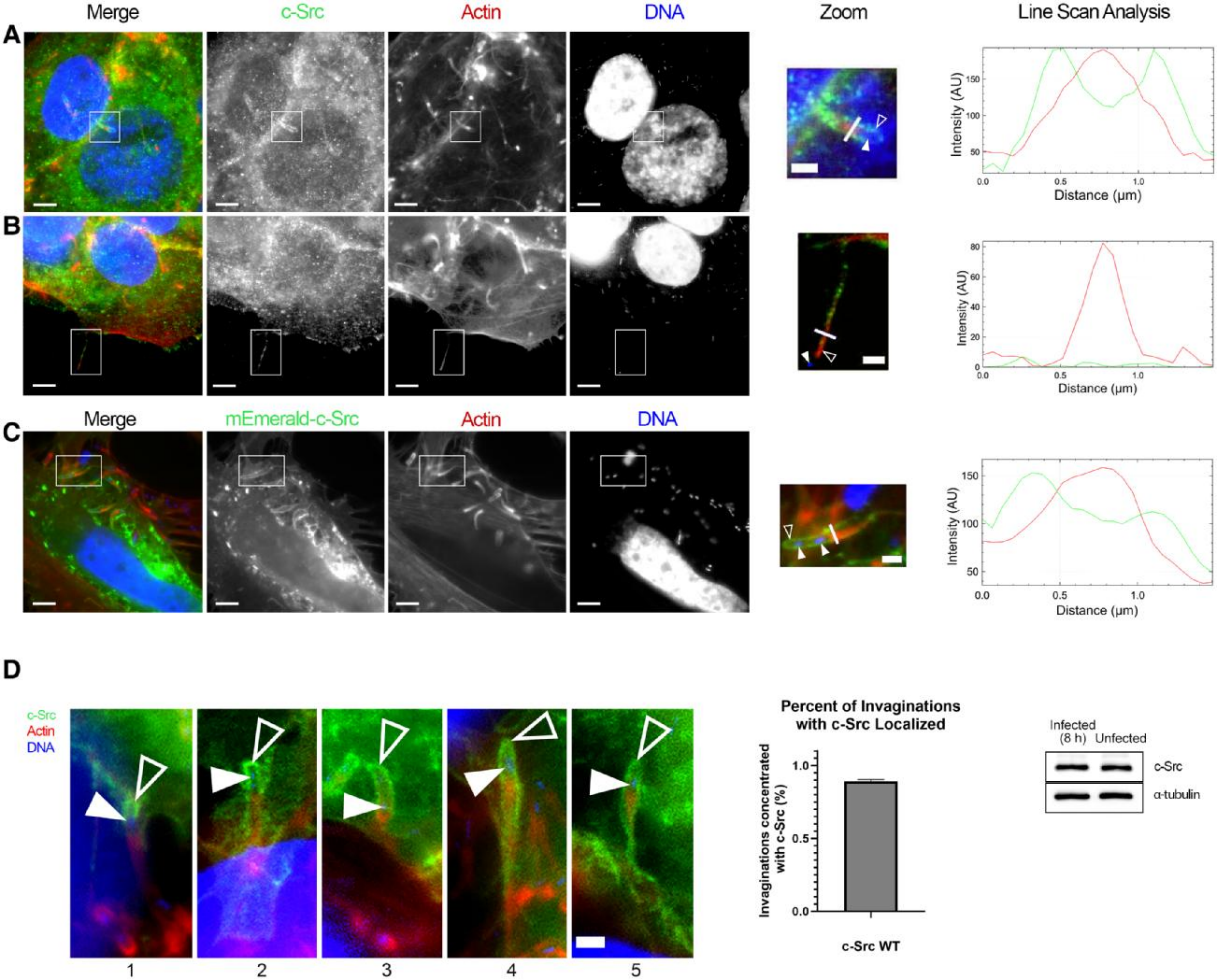
Localization of c-Src during *Listeria monocytogenes* infections. *A*, Jeg-3 cells infected with *L. monocytogenes* and stained with c-Src–targeting antibodies show increased protein localization at invaginations. *B*, Cells under the same infection protocol illustrating the lack of c-Src localization at listeriopods. *C*, Jeg-3 cells transfected with mEmerald c-Src and infected with *L. monocytogenes* show increased protein localization at invaginations. Scale bar = 5 µm. Zoomed insets of corresponding boxed regions for invaginations (*A* and *C*) and listeriopods (*B*) are shown. Scale bar = 1.5 µm. Line scan analysis of *L. monocytogenes* invaginations (*A* and *C*) and listeriopods (*B*) illustrates the relative intensity of actin and c-Src at the corresponding structures. This analysis was done by drawing a 1.5-µm line is across the bacterial structure and the F-actin (red) and c-Src protein (green) intensities are plotted. *D*, Localization of mEmerald c-Src at varying stages of *L. monocytogenes* invagination formation; increasing numbers correspond to later time points. Scale bar = 1.5 µm. The bar graph illustrates the percentage of invaginations that have c-Src localized during *L. monocytogenes* cell-to-cell spreading assays. This analysis was done with 3 individual replicates and at least 40 cells were included. In a western blot analysis, whole Jeg-3 cell lysates from uninfected and infected cells were probed for endogenous c-Src; α-tubulin is shown as a loading control. Solid arrows indicate spreading bacteria, and open arrows indicate invagination/listeriopod regions. Abbreviation: WT, wild type; AU, arbitrary units.

To investigate c-Src's role in *L. monocytogenes* cell-to-cell spreading, we immunolocalized c-Src in infected monolayers of Jeg-3 cells and found noticeably elevated levels of c-Src accumulated at sites of bacterial cell-to-cell spreading (Figure 1*A*). C-Src delineated the host plasma membrane encapsulating the protruding bacteria. To determine the precise localization of c-Src (at the listeriopods or corresponding invaginations) we examined listeriopods projecting into a cell-free space (Figure 1*B*). No obvious enrichment of c-Src above cytoplasmic levels was evident at listeriopods, indicating that c-Src localization during *L. monocytogenes* cell-to-cell spreading likely occurred at invaginations in the receiving cells.

To further characterize the accumulation of c-Src at invaginations, we performed line scan analysis to measure the protein intensity levels of c-Src with filamentous actin at cell-to-cell spreading sites. This analysis demonstrated c-Src expression at the invaginations, which lay peripheral to the actin-rich listeriopod as 2 green c-Src peaks (Figure 1*A* and 1*C* right), highlighting the increased expression of c-Src at invaginations surrounding the internalized listeriopod. Conversely, the opposite was true for listeriopods extending into empty space, where c-Src peaks were absent (Figure 1*B* right), highlighting the lack of protein expression at listeriopods. To further confirm c-Src localization at *L. monocytogenes* invaginations, host cells were transfected with mEmerald-tagged c-Src in receiving cells, which showed intense staining at the invagination sites (Figure 1*C*). We then sought to find out how frequently c-Src was recruited to *L. monocytogenes* invaginations. By performing cell-to-cell assays, we calculated the percent of invaginations with c-Src localization in the recipient cells. We found that c-Src localized to 89% of invaginations formed in the receiving cells. c-Src's localization at varying stages of invagination development were analyzed during cell-to-cell spreading assays. Figure 1*D*, left, demonstrates how c-Src localized continuously throughout the formation of invaginations from the beginning until complete uptake of *L. monocytogenes*. The increased expression of c-Src at the *L. monocytogenes* invaginations during the infections did not correspond to changes in endogenous c-Src levels when compared to uninfected Jeg-3 cells (Figure 1*D* right).

**Figure 2.**
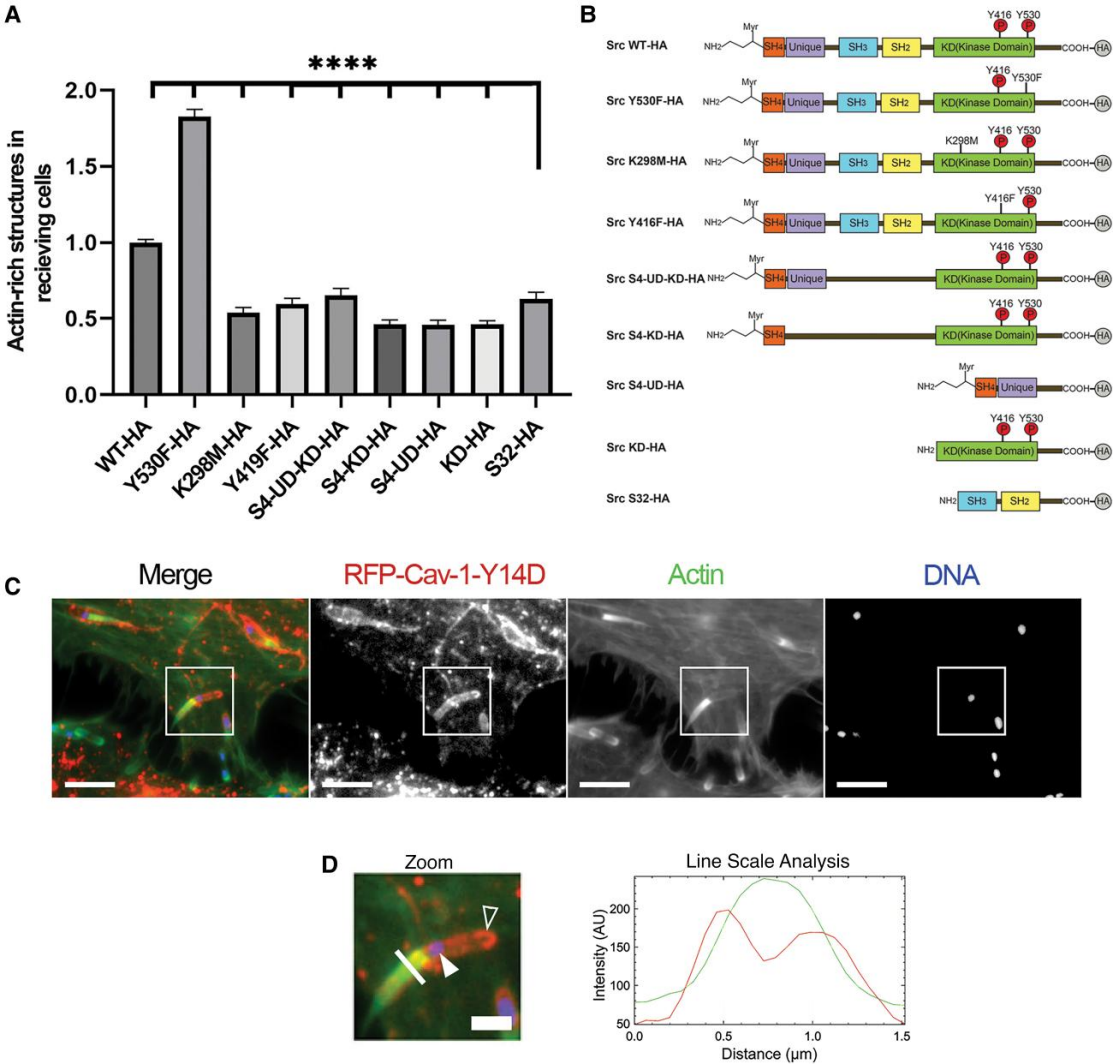
Cell-to-cell spreading assays with HA-WT and 8 HA-c-Src mutants. *A*, One-way ANOVA test comparing the number of actin-rich structures in mutant c-Src expressing Jeg-3 cells in comparison to controls (WT). At least 50 cells were counted and 3 individual trials were run. ****P < .01. *B*, Domain architecture of WT and the 8 mutants of c-Src. *C*, HeLa cav-1 KD cells transfected with RFP-cav-1-Y14D and infected with *Listeria monocytogenes* show increased localization at invaginations. Scale bar = 5 µm. *D*, Zoomed inset of boxed region. Scale bar = 1.5 µm. *D*, Line scan analysis of *L. monocytogenes* invaginations with F-actin and cav-1-Y14D intensity plotted. Solid arrow indicates spreading bacteria and open arrow indicates invagination regions. Abbreviations: UD, unique domain; WT, wild type; HA, hemagglutinin tagged; RFP, red fluorescence protein; KD, kinase domain.

To functionally assess the role of c-Src during *L. monocytogenes* cell-to-cell spreading, we performed cell-to-cell spreading assays where wild-type Jeg-3 cells that were initially infected with *L. monocytogenes* were mixed with uninfected cells (receiving cells) that expressed either wild-type or 8 different HA-tagged mutant forms of c-Src. This included point mutations (Y530F and K298M, respectively) that directly influenced c-Src's ability to phosphorylate its targets, as well as domain deletions known to affect the overall function of c-Src (Figure 2*B*). Bacterially associated actin-rich structures (actin clouds, comet tails, and listeriopods) were counted in the receiving c-Src–expressing cells as a measure of cell-to-cell spreading. C-Src Y530F has a single point mutation at tyrosine 530 changed to phenylalanine, making this protein constitutively active [12]. We found that cells expressing that construct had over 50% more actin-rich structures in the receiving cells than cells expressing wild-type c-Src. K298M is known to be constitutively inactive [12]. We found that receiving cells expressing that mutant had significantly fewer actin-rich structures compared to wild-type expressing cells. Another point mutation investigated was c-Src Y419F. This mutation affects the ability of c-Src to dimerize (dimerization is required for its kinase activity) [12]. Cells expressing this mutant also had approximately 50% fewer actin-rich structures compared to wild-type expressing cells (Figure 2*A*).

In general, cells expressing the various domain deletion mutants that impacted c-Src's ability to phosphorylate its targets had significantly fewer actin-rich structures. This included c-Src S4-UD-KD, which had a decrease of 35% in *L. monocytogenes*-associated actin-rich structures; c-Src S4-KD, which had 62% fewer actin-rich structures; c-Src S4-UD, having 64% fewer actin-rich structures; c-Src S32, which had 46% fewer bacterially associated actin-rich structures; and c-Src KD, which had 66% fewer actin-rich structures (Figure 2*A*).

With c-Src accumulating at *L. monocytogenes* invaginations and affecting cell-to-cell spreading, we sought to determine whether caveolin-1, the only known c-Src–activated protein at invagination sites, could be present in a phosphorylated form. To do this, we transfected a phosphomimicking mutant of caveolin-1 (caveolin-1 with a single point mutation from tyrosine to aspartate to act as a constitutively phosphorylated form of caveolin-1 [cav-1-Y14D]) into cav-1 stably KD HeLa cells to examine its localization. Figure 2*C* shows cav-1-Y14D concentrated at *L. monocytogenes* invaginations. An intensity graph was plotted to illustrate the accumulation of cav-1-Y14D at the sites (Figure 2*D*) with similar protein intensity results as the c-Src line scan analysis.

## DISCUSSION

*L. monocytogenes* cell-to-cell spreading is a crucial step towards the bacteria occupying entire organs during infection. Despite its importance, very few proteins have been identified at *L. monocytogenes* invaginations. These include those associated with caveolin-mediated endocytosis (caveolin-1, cavin-2, dynamin 2, epsin-1, EHD2), the formin mDia-1, CD147, myosin 1C and filamin A, E-cadherin, and actin [7, 8, 13–15]. Of those proteins, only caveolin-1 and dynamin-2 are known to be phosphorylated by c-Src [10, 11]. Caveolin-1 requires c-Src–mediated phosphorylation of Y14 to activate caveolin [11] and as both proteins are concentrated at the same sites it is expected that this is one of Src's targets.

Cell-to-cell spreading assays showed how c-Src mutants that render caveolin constitutively active cause an increase in *L. monocytogenes* spreading. Conversely, cells expressing c-Src that was unable to phosphorylate caveolin-1 (through single point mutations or domain deletions) had decreased cell-to-cell spreading. The increased expression of c-Src at *L. monocytogenes* invaginations together with the cell-to-cell spreading assay results suggest a functional role of c-Src at the invaginations formed in the receiving cells. A proposed model of c-Src phosphorylating caveolin-1 to support the cell-to-cell spread of *L. monocytogenes* is shown in Supplementary Figure 1. How might c-Src phosphorylation increase caveolin-mediated endocytosis? Zimnicka and colleagues found that c-Src–dependant phosphorylation of caveolin-1 Tyr-14 induced a conformational change that loosens caveolin-1 molecules in the caveolar complex to increase the number and size of the vesicles endocytosed [11]. We suggest that this is also a contributing mechanism at play during *L. monocytogenes* cell-to-cell spreading. There are likely additional proteins at *L. monocytogenes* invaginations that remain unidentified, which could also be phosphorylated by c-Src. Thus, characterizing proteins at the invaginations and assaying their potential for c-Src–based tyrosine phosphorylation remains an avenue for future exploration.

## Supplementary Material

jiaf063_Supplementary_Data

## Supplementary Data

Supplementary materials are available at *The Journal of Infectious Diseases* online (http://jid.oxfordjournals.org/). Supplementary materials consist of data provided by the author that are published to benefit the reader. The posted materials are not copyedited. The contents of all supplementary data are the sole responsibility of the authors. Questions or messages regarding errors should be addressed to the author.

## Notes

***Acknowledgments.*** We thank Pascale Cossart for providing the *Listeria* EGD BUG 600 wild-type strain and Robert Nabi for the caveolin-Y14D construct. Plasmids were gifted to us from Mark Moasser (Addgene plasmid numbers: 140294, 140296, 140300, 140304, 140312, 140314, 140316, 140318, and 140308).

***Financial support.*** This work was supported by the Canadian Institutes of Health Research (grant number 178189).

## References

[1] Radoshevich L, Cossart P. *Listeria monocytogenes*: towards a complete picture of its physiology and pathogenesis. Nat Rev Microbiol 2018; 16:32–46. 29176582 10.1038/nrmicro.2017.126

[2] Dramsi S, Biswas I, Maguin E, et al Entry of *Listeria monocytogenes* into hepatocytes requires expression of inIB, a surface protein of the internalin multigene family. Mol Microbiol 1995; 16:251–61. 7565087 10.1111/j.1365-2958.1995.tb02297.x

[3] Gaillard JL, Berche P, Frehel C, Gouin E, Cossart P. Entry of *L. monocytogenes* into cells is mediated by internalin, a repeat protein reminiscent of surface antigens from gram-positive cocci. Cell 1991; 65:1127–41. 1905979 10.1016/0092-8674(91)90009-n

[4] Veiga E, Guttman JA, Bonazzi M, et al Invasive and adherent bacterial pathogens co-opt host clathrin for infection. Cell Host Microbe 2007; 2:340–51. 18005755 10.1016/j.chom.2007.10.001 PMC2803069

[5] Kocks C, Gouin E, Tabouret M, Berche P, Ohayon H, Cossart P. *L. monocytogenes*-induced actin assembly requires the actA gene product, a surface protein. Cell 1992; 68:521–31. 1739966 10.1016/0092-8674(92)90188-i

[6] Sechi AS, Wehland J, Small JV. The isolated comet tail pseudopodium of *Listeria monocytogenes:* a tail of two actin filament populations, long and axial and short and random. J Cell Biol 1997; 137:155–67. 9105044 10.1083/jcb.137.1.155 PMC2139863

[7] Dhanda AS, Yu C, Lulic KT, et al *Listeria monocytogenes* exploits host caveolin for cell-to-cell spreading. mBio 2020; 11:e02857-19. 31964732 10.1128/mBio.02857-19 PMC6974566

[8] Sanderlin AG, Vondrak C, Scricco AJ, Fedrigo I, Ahyong V, Lamason RL. RNAi screen reveals a role for PACSIN2 and caveolins during bacterial cell-to-cell spread. Mol Biol Cell 2019; 30:2124–33. 31242077 10.1091/mbc.E19-04-0197 PMC6743452

[9] Boggon TJ, Eck MJ. Structure and regulation of Src family kinases. Oncogene 2004; 23:7918–27. 15489910 10.1038/sj.onc.1208081

[10] Shajahan AN, Timblin BK, Sandoval R, Tiruppathi C, Malik AB, Minshall RD. Role of Src-induced dynamin-2 phosphorylation in caveolae-mediated endocytosis in endothelial cells. J Biol Chem 2004; 279:20392–400. 15007081 10.1074/jbc.M308710200

[11] Zimnicka AM, Husain YS, Shajahan AN, et al Src-dependent phosphorylation of caveolin-1 tyr-14 promotes swelling and release of caveolae. Mol Biol Cell 2016; 27:2090–106. 27170175 10.1091/mbc.E15-11-0756 PMC4927282

[12] Spassov DS, Ruiz-Saenz A, Piple A, Moasser MM. A dimerization function in the intrinsically disordered N-terminal region of Src. Cell Rep 2018; 25:449–63.e4. 30304684 10.1016/j.celrep.2018.09.035 PMC6226010

[13] Dhanda AS, Lulic KT, Yu C, Chiu RH, Bukrinsky M, Guttman JA. *Listeria monocytogenes* hijacks CD147 to ensure proper membrane protrusion formation and efficient bacterial dissemination. Cell Mol Life Sci 2019; 76:4165–78. 31076805 10.1007/s00018-019-03130-4 PMC11105388

[14] Dhanda AS, Vogl AW, Ness F, Innocenti M, Guttman JA. Mdia1 assembles a linear F-actin coat at membrane invaginations to drive *Listeria monocytogenes* cell-to-cell spreading. mBio 2021; 12:e0293921. 34781738 10.1128/mBio.02939-21 PMC8593688

[15] Radhakrishnan P, Sathe M, Theriot JA. *Listeria monocyt*ogenes co-opts caveolin-mediated E-cadherin trafficking and macropinocytosis for epithelial cell-to-cell spread. bioRxiv, doi: 10.1101/2022.04.06.487361, 6 April 2022, preprint: not peer reviewed.

