# Supplementary material for: Characterization of c-Src During Listeria monocytogenes Cell-to-Cell Spreading
Source: J Infect Dis. 2025 Feb 3;231(5):e912–7. doi: 10.1093/infdis/jiaf063 (PMC12128067; doi:10.1093/infdis/jiaf063)
Supplement: jiaf063_Supplementary_Data [file jiaf063_supplementary_data.docx]

**Supplementary**

Methods

Paraformaldehyde was prepared in 150 mM NaCl, 4 mM Na/K PO_4_, 5.0 mM KCl, pH 7.3.

RIPA lysis buffer consisted of 150 mM NaCl, 50 mM Tris [pH 7.4], 5 mM EDTA, 1% Nonidet P-40, 1% deoxycholic acid, 10% SDS.

Mild stripping buffer included 1.5% glycine, 0.1% SDS, 1% Tween 20, pH 2.2. TPBS/BSA was made using PBS[-/-], 0.5% Tween 20, 0.1% bovine serum albumin (BSA).

TBST consisted of Tris-buffered saline, 0.05% Tween 20.

TPBS/BSA consisted of PBS[-/-], 0.5% Tween 20, 0.1% bovine serum albumin.


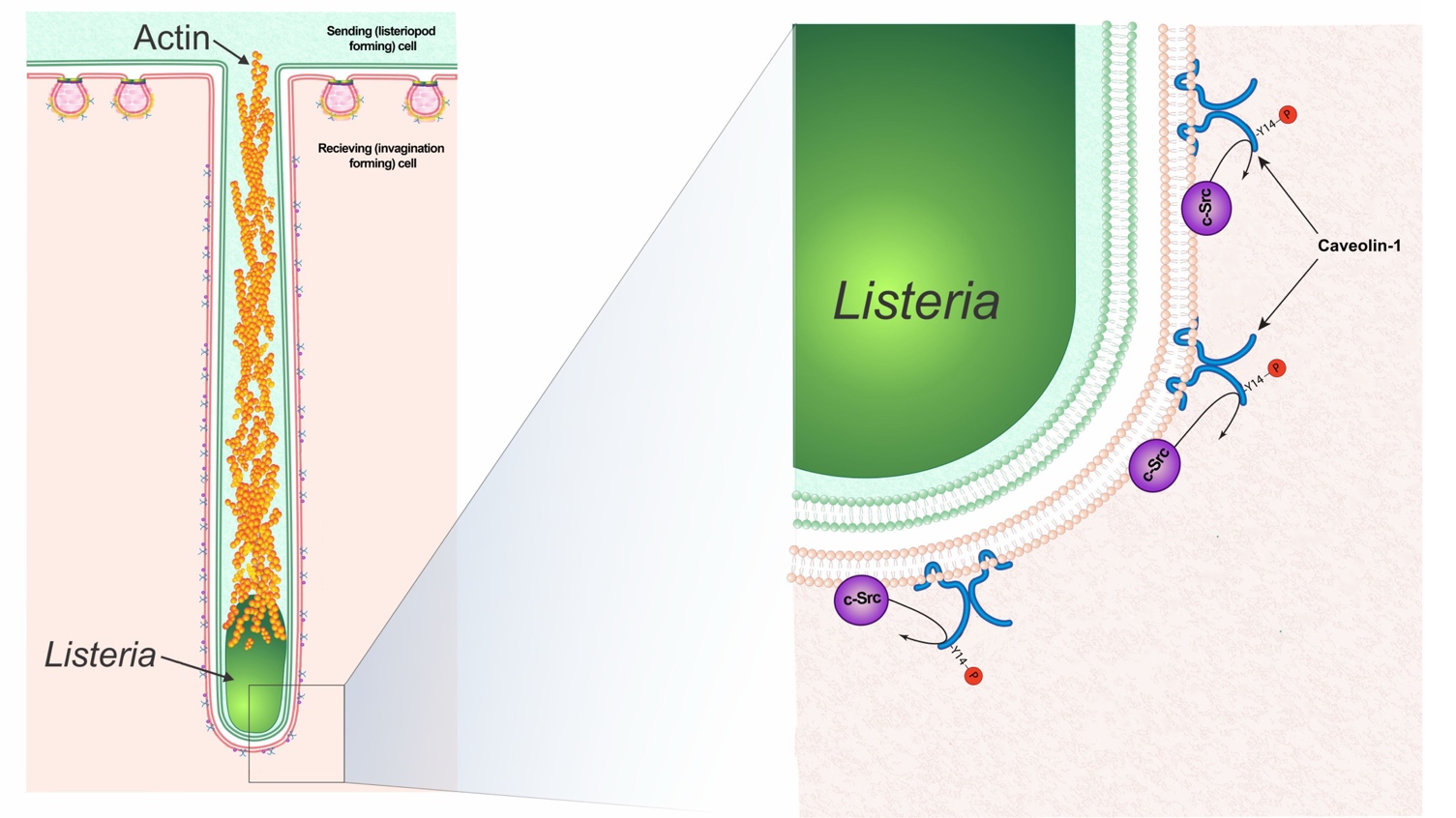


Figure S1. Proposed model of c-Src phosphorylating caveolin-1 to trigger the formation of invaginations (modified from [12])*.* We propose that c-Src binds to the membrane to phosphorylate caveolin-1 at tyrosine 14 to drive the uptake of *L. monocytogenes* into receiving cells.
